# Exploring Orthopedic Occupational Therapy in Korea: Insights from a Survey on Occupational Therapists' Perspectives

**DOI:** 10.1155/2023/5566248

**Published:** 2023-09-08

**Authors:** Junghun Aj Kim

**Affiliations:** Research Center of Industry-Academic Cooperation Foundation, Yonsei University, Wonju 26493, Republic of Korea

## Abstract

This study explores the perceptions and experiences of Korean occupational therapists (OTs) about their role in managing elderly patients with orthopedic conditions. The goal is to inform policy discussions for better integration of OT services in orthopedic care settings in Korea. A survey was conducted among Korean clinical OTs to gather data on their perceptions, experiences, and challenges in providing orthopedic OT services. Snowball sampling was used, and the survey addressed general characteristics, orthopedic OT status, perceptions, and competence. The data were analyzed using frequency and percentage calculations in SPSS 22.0. Out of 171 respondents, only 18 had experience working in orthopedic departments, while 78 (45.6%) had provided occupational therapy to orthopedic patients. Rehabilitation medicine physicians were the primary prescribers of orthopedic OT. Key challenges included a lack of awareness among professionals, an absence of appropriate fees, and insufficient knowledge among OTs. The majority of respondents agreed that providing OT in orthopedic departments was appropriate and expressed a need for education and clinical guidelines. The study examines the current state of orthopedic OT in Korea, showing low levels of experience and highlighting challenges, such as a lack of professional awareness, inadequate fees, and insufficient knowledge among OTs. Respondents mostly agreed on the necessity for education and clinical guidelines to improve their capabilities in orthopedic settings. This study emphasizes the need for healthcare system improvements that allow OTs to participate more widely without being confined to specific medical disciplines, promoting a more comprehensive approach to OT, especially as the aging population continues to grow.

## 1. Introduction

Orthopedic conditions, including degenerative joint diseases, fractures, and musculoskeletal disorders, are prevalent among the elderly population, posing significant challenges to their functional independence and quality of life [1]. As the aging population continues to grow rapidly in Korea, the demand for comprehensive and patient-centered care for individuals with orthopedic problems is expected to rise [2, 3].

In response to this growing need, the number of registered occupational therapists in Korea has been on the rise. As of 2023, there are 19,136 registered occupational therapists, equating to about 38.8 therapists per 100,000 population [4]. This is a notable increase from the 29 per 100,000 population reported in 2017. These professionals predominantly find employment in hospitals and community practices. Regarding the aging population, it is noteworthy that the expansion of the occupational therapy (OT) area has been primarily directed towards cognitive domains, such as dementia centers or daycare centers in communities, which are covered by the government's long-term care insurance [5].

Occupational therapists (OTs) play a vital role in the interdisciplinary care team for patients with orthopedic conditions, providing valuable interventions to help them regain functional independence and improve their overall well-being [2, 3]. However, the Korean healthcare system faces challenges in incorporating OTs in orthopedic care settings due to governmental healthcare policies [6]. Consequently, Korean Orthopedic Specialty Hospital rarely employs OTs, potentially affecting the quality of rehabilitation services provided to the elderly with orthopedic conditions. As a result, in the case of lower extremity joint replacement surgeries, which are the most common among elderly Koreans, the average length of hospital stay is significantly longer at approximately 20 days compared to developed countries [6, 7].

This study is aimed at exploring Korean OTs' perceptions of their role and the scope of orthopedic OT in the management of elderly patients with various orthopedic problems. Through the use of a survey, we gathered information about Korean OTs' awareness, perceptions, and experiences in providing orthopedic OT services to elderly patients. Our findings will contribute to a better understanding of the challenges, opportunities, and potential areas of improvement in the delivery of orthopedic OT for elderly patients in Korea and inform discussions on policy changes to better integrate OT services into orthopedic care settings.

## 2. Methods

### 2.1. Research Subjects and Data Collection

This study was conducted following the research approval granted by the Yonsei University Mirae Campus Institutional Review Board (1041849-202006-BM-070-01). This survey study is part of the process of the “development of occupational therapy practice guidelines for patients with total knee replacement.”

To conduct the survey, clinical OTs working in Korea were recruited using snowball sampling from May 2nd to June 21st, 2022. Snowball sampling was chosen due to the specific nature of our target population. Clinical OTs specializing in orthopedic care might represent a specialized subset within the broader OT community, making them harder to locate through conventional sampling methods. This method capitalizes on existing networks and connections among professionals, ensuring more comprehensive access to potential participants with the desired expertise. The recruitment process was facilitated through social media groups in which general OTs participated. The Google Forms survey URL was sent to those who voluntarily agreed to participate after reading the research information and signing consent forms.

Before initiating the survey, we set specific criteria to select our participants. To qualify for inclusion in our study, participants had to (1) hold a valid South Korean OT license, (2) have work experience of more than 1 year, and (3) be currently employed in any occupational therapy practice. Conversely, the sole exclusion criterion was that occupational therapists who were not currently practicing as OTs could not participate.

### 2.2. Materials

The survey was composed of sections addressing the general characteristics of participants, the status of orthopedic OT, perceptions of OTs, and topics related to their competence in dealing with people with musculoskeletal disorders. It was based on Causey-Upton et al.'s (2018) survey targeting OT [8]. In General Characteristics, 6 questions were included, covering career and field. Orthopedic Occupational Therapy Experience had 4 questions, and Perceptions of OT for Patients with Orthopedic Surgery had 5 questions. Responses were formatted as either a Yes or No choice or a 4-point Likert scale, consisting of “Strongly Disagree,” “Disagree,” “Agree,” and “Strongly Agree.”

### 2.3. Data Analysis

Data were analyzed using the SPSS 22.0. Statistics were used for data analysis to determine the frequency and percentages of responses on survey items. For the perception of OT for patients with orthopedic surgery questions, frequency analysis and percentage calculation were conducted. After that, responses for “agree” and “strongly agree” under the category of “perception” were grouped together as “yes” for analysis of the proportion according to the respondent's general characteristics, which was then represented in a graph.

## 3. Results

### 3.1. General Characteristics

A total of 171 individuals responded to the survey. Of these, 65 (38.0%) were male and 106 (62.0%) were female. The average age of the respondents was 26.9 ± 7, with a minimum age of 21 and a maximum age of 56. The age group with the highest response rate was 21-25 years old (40.7%). The respondents' clinical experience averaged 5.9 ± 5.9 years, ranging from a minimum of 1 year to a maximum of 37 years. The largest proportion of respondents, 50.6%, had 1-3 years of experience. In terms of educational level, 52.6% of respondents held a university degree. Geographically, 110 respondents (64.3%) were from Seoul, Gyeonggi-do, and Incheon. Regarding practice settings, rehabilitation hospitals were the most common, with 78 respondents (45.6%) (Table 1).

### 3.2. Orthopedic Occupational Therapy Experience

Of the respondents, 18 (10.5%) OTs with experience working in orthopedic departments participated in the survey. A total of 78 respondents (45.6%) indicated that they had provided OT to orthopedic patients (Table 2).

In response to the question about which physicians prescribed OT for orthopedic patients, 74.7% of the respondents indicated that it was rehabilitation medicine physicians, confirming a significantly higher proportion in this specialty. Additionally, the types of orthopedic patients included lower joint replacement surgeries and back disorders at 45.1%, followed by 18.0% with arthritis (Table 3).

When asked about the reasons for the difficulty in providing OT in orthopedic departments, the OTs cited the highest cause as “Lack of awareness among other professionals,” with 125 respondents (25.4%). This was followed by “Absence of appropriate fees,” with 110 respondents (22.3%). Moreover, “Lack of knowledge and awareness among occupational therapists” also had a high response rate with 108 respondents (21.9%) (Table 4).

### 3.3. Perception of OT for Patients with Orthopedic Surgery

The following responses pertain to perceptions of OT in orthopedic departments, with questions asked using a 4-point Likert scale. Among the respondents, 163 (95.3%) indicated that providing OT in orthopedic departments was appropriate. Regarding their “Level of knowledge in orthopedic occupational therapy,” 94 respondents (55.0%) agreed that they had sufficient knowledge, while 77 (45.0%) disagreed, citing a lack of knowledge and experience. The responses for “Intention to participate in orthopedic OT education” and “The necessity of clinical guidelines for orthopedic occupational therapy” were predominantly “agree,” with 153 (89.5%) and 168 (98.2%) respondents, respectively. “Intention to work in orthopedic surgery” was reported by 105 respondents (61.4%) (Table 5).

The awareness of orthopedic OT among the responding OTs was examined separately according to their characteristics. On the 4-point scale, “Agree” and “Strongly agree” responses were combined as “Yes” and compared with general characteristics (Table 6). No significant differences were observed in perceptions based on gender. The level of knowledge about orthopedic OT tended to increase with age and was particularly high among those with a doctorate, at 94.7%. OTs working in community settings also reported higher knowledge levels (80%). The need for education and guidelines on orthopedic OT was high across all strata, with a higher demand for guidelines. In contrast, the “intention to work in orthopedic surgery (OS)” had the highest positive response rate among respondents aged 41 and older (80.0%), was highest at the college education level (74.4%), and was most prominent among those working in rehabilitation hospitals (80%).

## 4. Discussion

This survey study was conducted to investigate the current state of orthopedic OT and the perceptions of OTs during the research process of “Development of Occupational Therapy Practice Guidelines for Patients with Total Knee Replacement.”

Participants were sampled using the snowball method, and responses were collected from a total of 171 OTs through the implementation of the survey. In Korea, forming and communicating within OT communities through social media is a prevalent trend. Consequently, the survey URL was shared within social media OT communities to gather responses. As a result, the participation rate of young, active social media users in their 20s was notably high, accounting for over 60% of the OTs involved in the study.

In terms of regional characteristics, over 60% of the responses were from Seoul, Gyeonggi-do, and Incheon, where 50% of the population is concentrated. In contrast, areas with smaller populations, such as Gangwon-do and Jeju-do, showed lower response rates, with 6 (3.5%) and 4 (2.3%), respectively. As for practice settings, rehab hospitals, where the majority of OTs in Korea work, were most prevalent [5], a finding consistent with other survey studies [9].

The investigation into the current state of orthopedic OT revealed that “orthopedic occupational therapist work experience” was relatively low at approximately 10%, while the experience of providing OT for orthopedic patients was higher at 45.6%. This result confirms that orthopedic patients can be encountered in various settings.

In this study, it was found that OT prescriptions for orthopedic surgery patients were primarily made by rehabilitation medicine departments (74.7%). This is likely due to the fact that in the current Korean healthcare system, OT prescriptions are limited outside of rehabilitation medicine [10]. Consequently, patients not affiliated with rehabilitation medicine departments can only receive OT if they are referred to such a department. It is important to highlight that patients who undergo orthopedic surgery often move to rehabilitation hospitals for postsurgical rehabilitation before being discharged home. This transition ensures they receive the necessary OT services to prepare for activities of daily living. Without this affiliation to rehabilitation medicine departments, many patients might be discharged home postsurgery without adequate preparation for their daily life tasks, potentially compromising their recovery and functional independence.

This limitation makes it challenging for patients with conditions or disabilities not treated in rehabilitation medicine departments to access OT. Consequently, patients with chronic diseases such as diabetes, hypertension, cardiovascular disorders, or those who develop disabilities due to aging may require OT but are unable to receive it [6].

When asked about the reasons for the difficulty in applying OT to orthopedic patients, the most common response (25.4%) was “lack of awareness among other professionals.” This corresponds with the perception that the healthcare community in Korea has had a low level of awareness about OT [11, 12]. This could be due to the relatively short history of OT in the country and the small number of OTs. However, it might be due to the absence of interactions with other health fields resulting from the system that restricts prescriptions and the implementation of OT in rehabilitation medicine departments. Additionally, as OTs pointed out, the “absence of appropriate fees” could also be a contributing factor.

Currently, besides rehabilitation medicine doctors, orthopedic surgeons, neurosurgeons, and neurologists can prescribe OT. However, for domains other than rehabilitation medicine, the prescription authority is categorized into “simple occupational therapy” and “complex occupational therapy” items [13]. Specifically, “simple occupational therapy” refers to a situation where one occupational therapist conducts training for more than two patients simultaneously for a duration of at least 10 minutes. On the other hand, “complex occupational therapy” denotes a scenario where one occupational therapist focuses on one patient in a one-on-one setting for a duration of roughly 10 to 30 minutes. The fees associated with these services are relatively low, leading to limited prescriptions by doctors and a reduced employment rate of OTs [6].

Another reason cited was the “lack of knowledge and awareness among occupational therapists,” as reported by 108 respondents (21.9%). The results show that even among those with a practice period of 1-3 years, 56.3% claim to have orthopedic knowledge and experience. This demonstrates that clinical experience does not significantly increase with time. Although subjects related to orthopedics are taught in university curricula, it is difficult to meet related patients in practice. As time goes by, therapists may find their competence with patients to be insufficient. This lack of competence can be observed in the high demand for orthopedic education and clinical guidelines, as seen in the results.

Most OTs believe that it is appropriate for them to work in orthopedic specialty hospitals (Table 5). However, 44.4% of them feel unconfident about their knowledge of orthopedic OT. This is further reflected in their willingness to participate in orthopedic OT education, with approximately 89.5% of OTs expressing their interest. Moreover, 98.2% of OTs responded that clinical guidelines applicable in practice would be helpful.

To address these concerns, it is essential to look beyond the clinical guidelines. Continuous professional development through specialized workshops, mentorship programs, on-site training at orthopedic specialty hospitals, active research engagement, and the establishment of centralized resource centers can play pivotal roles. Additionally, instituting feedback mechanisms where therapists can receive feedback on their strategies in handling orthopedic cases can help refine their practices. In conclusion, it is argued that improvements in the healthcare system that allow OTs to participate without being confined to specific medical disciplines are necessary for a more comprehensive approach to OT.

OT inherently plays a crucial role in patients' rehabilitation through a team approach, particularly in the rehabilitation and home return of the elderly, as it significantly impacts their daily lives. OTs are needed for discharge planning after joint replacement surgeries or back disorder surgeries stemming from age-related degenerative conditions. However, in Korea, the system makes it difficult for OTs to directly receive prescriptions and treat patients in the field of orthopedics. As a result, the average length of hospital stays for total knee replacement (TKR) and total hip replacement (THR) patients is 21.1 days and 18.6 days, respectively, ranking them first and second for the longest hospital stays following surgery [7].

In detail, TKR, which ranks second in frequency among surgeries for the elderly in Korea, has varying hospital stay durations depending on the scale of the hospital: 13.56 days for tertiary hospitals, 23.10 days for general hospitals, 22.48 days for smaller hospitals, and 20.34 days for clinics [7]. The length of hospital stays at tertiary hospitals is about 10 days shorter than those at other institutions. However, even the hospital stay duration at tertiary hospitals is considerably longer compared to acute care hospitals in developed countries. When examining the average length of hospital stays in developed countries, the United Kingdom had 5.4 days [14], Canada had 4.4 days in 2012 [15], and the United States had 4.7 days in 2019 [16]. Even when compared to the shortest hospital stays at tertiary hospitals in Korea, there is a significant difference.

In multidisciplinary team settings, OTs play a crucial role in reducing the length of hospital stays. Gleicher et al. [17] conducted a pre-post comparative study to examine the effects of the enhanced recovery program (ERP) after surgery. The conventional care model without the involvement of OTs was applied before the intervention, while in the postintervention phase, the team identified the need for specialized involvement and included OTs in providing interventions during the patient's discharge planning process. As a result, the intervention group with OTs showed a reduced length of hospital stay (from 2.82 days to 2.13 days) and fewer emergency room visits. The study confirmed that OTs could contribute to stable early discharges by evaluating and intervening in patients' readiness for discharge and return to daily living during the recovery process.

Therefore, in Korea, as the number of elderly individuals undergoing musculoskeletal surgeries increases, improvements in the system that allow the provision of OT in orthopedics and efforts to strengthen the capabilities of OTs in related fields are necessary for the rapid and stable recovery of these patients.

There are some limitations to this study. First, the responses were collected through social media, which may have led to a higher response rate from younger OTs and the possibility of biased opinions being reflected. Second, an in-depth investigation of OTs working in orthopedic specialty hospitals was not conducted. The original plan was to interview these therapists, but it was found that most of them worked in general hospitals, and no OTs working in orthopedic specialty hospitals could be located. This suggests that OTs may not be employed in orthopedic specialty hospitals in Korea, which is a point to consider for future research. This research offers several significant strengths that enrich the understanding of orthopedic OT in Korea. Firstly, it provides a comprehensive exploration of a topic that has been relatively underrepresented in existing literature. This serves to bridge the knowledge gap, fostering a deeper understanding of the intricacies of orthopedic OT within the specific cultural and healthcare context of Korea. Secondly, by highlighting the prevailing gaps and needs, this study sets a foundation for policy recommendations, potential curriculum changes, and the direction of future research in this area.

## 5. Conclusion

This survey was conducted as part of the “Development of Occupational Therapy Practice Guidelines for Patients with Total Knee Replacement” research project, aiming to investigate the current state of orthopedic OT and the perceptions of OTs. It was found that very few OTs work in orthopedic settings. Nevertheless, it was confirmed that OTs have provided services to elderly patients with musculoskeletal disorders. Moreover, the majority of OTs emphasized the need for education and guidelines to enhance their capabilities in orthopedic settings.

In Korea, due to healthcare system issues, it is challenging to implement OT in fields other than rehabilitation medicine. With the increasing elderly population and the high frequency of orthopedic surgeries in this demographic, it is crucial to improve the system in this area to facilitate early discharges and successful returns to home life. This will help prevent healthcare cost losses for the elderly population and maintain their quality of life after discharge.

The OT guidelines for TKR patients, which will be developed based on this study, can be used to enhance the capabilities of OTs in orthopedic OT.

## Figures and Tables

**Table 1:** The general characteristics (*N* = 171).

Characteristics	Categories	*n*	(%)
Sex	Male	65	38.0
	Female	106	62.0

Age	21-25	70	40.7
	26-30	44	25.6
	31-35	18	10.5
	36-40	30	17.4
	over 40	10	5.8

Practice experience (year)	1-3	87	50.6
	4-6	25	14.5
	7-9	17	9.9
	10-12	15	8.7
	over 12	27	15.7

Education level	College	39	22.8
	University	90	52.6
	Master	22	12.9
	Doctorate	20	11.7

Region	Seoul	47	27.5
	Gyeonggi-do, Incheon	63	36.8
	Gangwon-do	6	3.5
	Chungcheong-do, Sejong-si, and Daejeon	22	12.9
	Jeolla-do, Gwangju	10	5.8
	Gyeongsang-do, Daegu, Ulsan, and Busan	19	11.1
	Jeju-do	4	2.3

Practice setting	General hospital	12	7.0
	Rehab. hospital	78	45.6
	Community public institutions	10	5.8
	Child center	25	14.6
	Elderly care-center	11	6.4
	Government office	4	2.3
	Community welfare center	4	2.3
	Other	27	15.8

**Table 2:** Orthopedic OT experience.

Experience	Response	
	Yes (%)	No (%)
Orthopedic occupational therapist work experience	18 (10.5)	153 (89.5)
Providing occupational therapy experience for orthopedic patients	78 (45.6)	93 (54.4)

**Table 3:** Prescribing OT service and type of OS patients.

Items	Responses	Number	%
Prescribing special list for OS patients	Department of Rehabilitation Medicine	62	74.7%
	Department of Neurosurgery	7	8.4%
	Department of Orthopedic Surgery	4	4.8%
	General practitioner	1	1.2%
	Not sure	9	10.8%

Types of OS patients receiving OT service (multiple responses)	THR, TKR	55	23.6%
	Back disorders	50	21.5%
	Arthritis	42	18.0%
	Fracture	26	11.2%
	Amputation	26	11.2%
	Hand injury	20	8.6%
	Pediatric musculoskeletal patients under 18 years old	14	6.0%

THR: total hip replacement; TKR: total knee replacement.

**Table 4:** Challenges in providing OT for OS patients.

Items	Responses	Number	%
Reasons for difficulty in providing occupational therapy in orthopedic hospitals	Absence of appropriate fees	110	22.3%
	Lack of knowledge and awareness among occupational therapists	108	21.9%
	Lack of awareness among other professionals	125	25.4%
	Difficulty in team approach for patient rehabilitation	29	5.9%
	Absence of clinical guidelines	72	14.6%
	No referral from orthopedic surgery to rehabilitation medicine	49	9.9%

**Table 5:** Perception of OT for patients with orthopedic surgery (*N* = 171).

Items	Responses			
	Strongly disagree	Disagree	Agree	Strongly agree
Is the provision of occupational therapy appropriate in orthopedic specialty hospitals?	3 (1.8)	5 (2.9)	73 (42.7)	90 (52.6)
Level of knowledge in orthopedic occupational therapy	11 (6.4)	66 (38.6)	71 (41.5)	23 (13.5)
Intention to participate in orthopedic occupational therapy education	6 (3.5)	12 (7.0)	85 (49.7)	68 (39.8)
The necessity of clinical guidelines for orthopedic occupational therapy	2 (1.2)	1 (0.6)	70 (40.9)	98 (57.3)
Intention to work in orthopedic surgery	17 (9.9)	49 (28.7)	75 (43.9)	30 (17.5)

**Table 6:** “Yes” ratio about OT perception by characteristics.

Characteristics (*n*)		Level of knowledge in orthopedic OT	Intention to participate in orthopedic OT education	The necessity of clinical guidelines for orthopedic OT	Intention to work in OS
		Yes ratio (%)	Yes ratio (%)	Yes ratio (%)	Yes ratio (%)
Sex	Male (65)	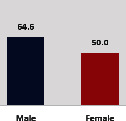	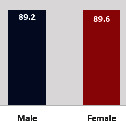	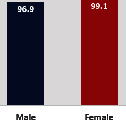	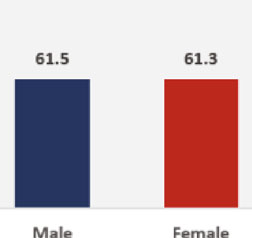
	Female (106)				

Age	21-25 (70)	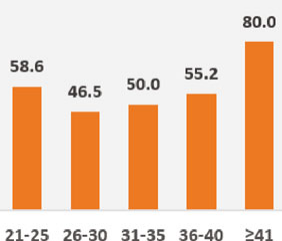	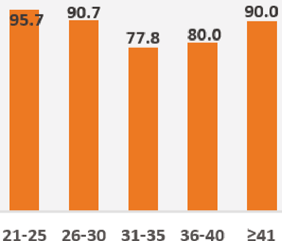	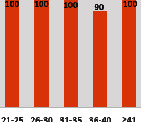	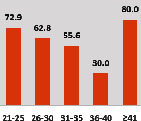
	26-30 (44)				
	31-35 (18)				
	36-40 (30)				
	Over 40 (10)				

Practice experience (year)	1-3 (87)	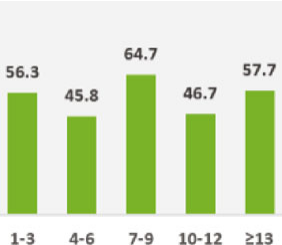	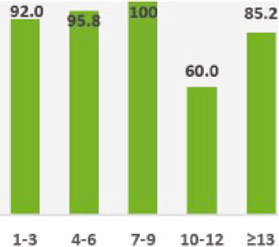	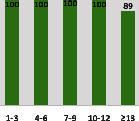	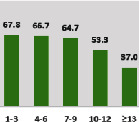
	4-6 (25)				
	7-9 (17)				
	10-12 (15)				
	Over 12 (27)				

Education level	College (39)	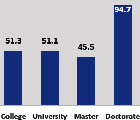	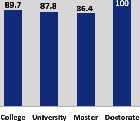	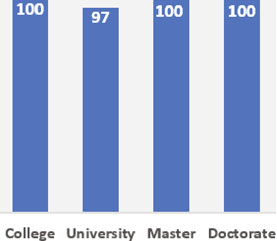	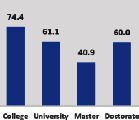
	University (90)				
	Master (22)				
	Doctorate (20)				

Practice setting	G. Hosp. (12)	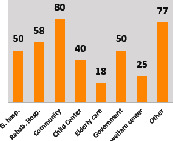	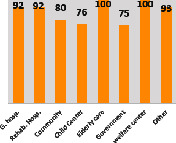	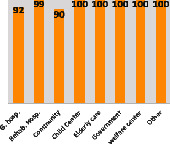	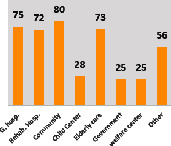
	Rehab. Hosp. (78)				
	Community (10)				
	Child Center (25)				
	Elderly care (11)				
	Government (4)				
	Welfare center (4)				
	Other (27)				

## Data Availability

The data used to support the findings of this study are available from the author upon request.
